# Gastric ectopic pancreas mimicking a gastrointestinal stromal tumour: A case report

**DOI:** 10.1016/j.ijscr.2018.11.014

**Published:** 2018-11-16

**Authors:** Zdravko Štor, Jurij Hanžel

**Affiliations:** Department of Abdominal Surgery, University Medical Centre Ljubljana, Zaloška 7, 1525 Ljubljana, Slovenia

**Keywords:** Stomach, Ectopic pancreas, Gastrointestinal stromal tumour, Laparoscopic excision

## Abstract

**Introduction:**

The diagnosis and management of upper gastrointestinal tract submucosal tumours can be challenging due to the difficulties with adequate pre-operative characterization and tissue sampling.

**Presentation of the case:**

A 61-year-old female patient underwent esophagogastroduodenoscopy because of epigastric pain and episodic vomiting. A submucosal tumour was found along the greater curvature in the antrum. On endoscopic ultrasound, the mass was suspected to be a gastrointestinal stromal tumour. The patient was referred for laparoscopic excision. The final histopathological examination confirmed an ectopic pancreas, not a gastrointestinal stromal tumour as thought preoperatively.

**Discussion:**

Despite an adequate pre-operative diagnostic workup with endoscopic ultrasonography, the lesion was misdiagnosed as a gastrointestinal stromal tumour and surgery was performed to excise an ectopic pancreas, essentially a benign lesion.

**Conclusion:**

The case highlights the absence of firm diagnostic criteria to differentiate submucosal gastric lesions.

## Highlights

• The diagnosis and management of gastric submucosal tumours are fraught with difficulty and uncertainty.
• After a thorough work-up with gastroscopy and endoscopic ultrasonography, a gastrointestinal stromal tumour of the antrum was diagnosed.
• Laparoscopic excision was performed – histopathologically, an ectopic pancreas was diagnosed.
• Despite an adequate work-up, surgery was performed for an essentially benign lesion.

## 1 Introduction

Submucosal tumours of the gastrointestinal tract are clinically characterized as protruding lesions, covered with intact mucosa [1]. Diagnosis using only esophagogastroduodenoscopy and endoscopic ultrasound remains difficult and many lesions are ultimately resected surgically [2]. The main diagnostic challenge in the stomach lies in distinguishing gastrointestinal stromal tumours (GIST) from benign lesions that do not require resection.

In the stomach, GIST are the commonest submucosal tumours. They develop from the interstitial cells of Cajal in the muscularis propria and are usually seen in the fourth layer of the gastric wall on endoscopic ultrasound [3]. Anatomically, 46.4–58% are found in the gastric body, 21–33% in the fundus, 13–18.9% in the antrum and 1.4–8% in the cardia [3]. Ectopic pancreas is pancreatic tissue without vascular or anatomical connection to the pancreas. On endoscopic ultrasound, they can be found in the third or fourth layer, within the stomach, the vast majority are found along the greater curvature in the antrum [3]. Malignant transformation is extremely rare [4].

The case is reported in accordance with the SCARE checklist [5].

## 2 Case presentation

**Fig. 1:**
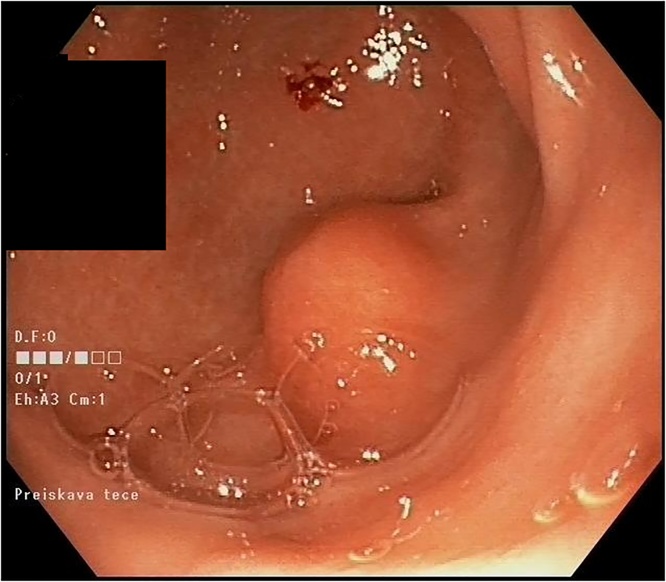
Endoscopic image of the gastric antral submucosal lesion, which ultimately proved to be a gastric ectopic pancreas.

A 61-year-old female patient was referred to our department for laparoscopic excision of GIST in the stomach. Prior to our treatment she underwent an esophagogastroduodenoscopy due to epigastric pain and occasional vomiting. Endoscopy showed erosive gastritis, a hiatal hernia, and a submucosal tumour located along the lesser curvature on the posterior wall of the antrum (Fig. 1). The tumour had a central eroded depression but otherwise appeared to be covered with normal mucosa. Several biopsies of the tumour were taken using the bite-on-bite technique. Unfortunately, only scant samples of the submucosa were obtained, which precluded a histological diagnosis of the tumour.

**Fig. 2:**
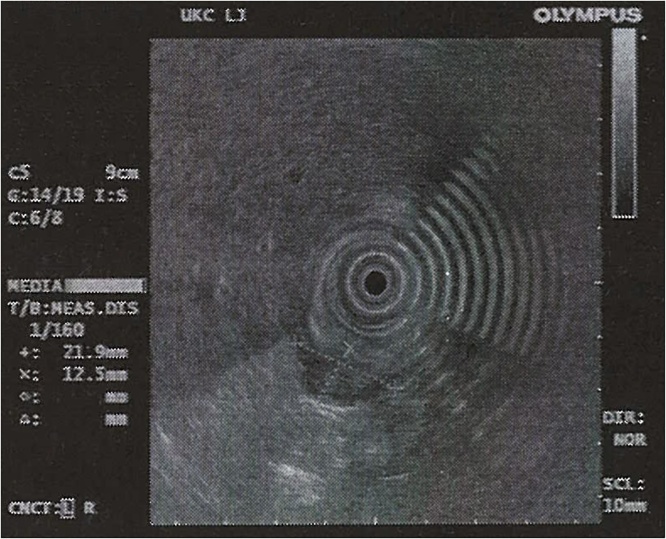
Endoscopic ultrasonography image of the gastric antral submucosal lesion, which ultimately proved to be a gastric ectopic pancreas. The lesion was growing from the fourth, muscular, layer of the stomach, measured 22 × 13 mm and displayed heterogenous echogenicity.

Endoscopic ultrasonography confirmed a 22 × 13 mm submucosal well-defined solid tumour arising from the fourth, muscular, layer of the stomach (Fig. 2). The tumour appeared to be nodular, with slightly heterogeneous echogenicity. Endoscopic ultrasonography did not demonstrate a significant ulceration or ductal structures. In the diagnostic work-up of abdominal pain and vomiting the patient had undergone transabdominal ultrasonography, which showed no focal lesions in the liver. Before surgery, we performed another endoscopy where the lesion was tattooed proximally and distally.

Laparoscopy was performed, and on initial exploration of the abdominal cavity, we discovered a tattooed submucosal tumour on the greater curvature of the stomach. Laparoscopic excision was performed and the specimen was recovered in a retrieval bag. The abdominal cavity was irrigated with saline and the fluid aspirated. An abdominal drain was inserted beneath the stomach. The fascia and skin wounds were sutured. The postoperative course was uneventful and the patient was discharged on the fifth postoperative day.

**Fig. 3:**
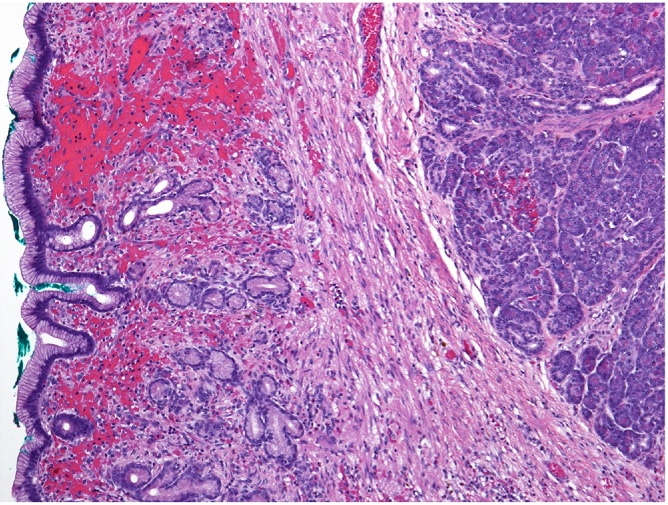
Ectopic pancreatic lobuli occupying the submucosa and muscularis propria under intact normal gastric mucosa (haematoxylin & eosin).

Resection samples were sent for histopathological analysis. The histopathologic analysis report about 6.6 × 3.5 cm excision gastric wall. The mucosa and serosa have normal appearance, muscularis propria and submucosa include a gray yellowish node 15 mm in diameter. The tumour did not involve the resection margins. Histopathological analysis confirmed an ectopic pancreas, 15 mm in diameter (Fig. 3).

## 3 Discussion

Ectopic pancreas is a congenital malformation of the pancreas, with an unclear pathogenesis – one of the postulated mechanisms is the separation and subsequent deposition at ectopic sites during rotation of the foregut [6,7]. Its frequency in autopsy series ranges from 0.5 to 13.7% [8]. Lesions are typically located in the jejunum, duodenum or stomach, the greater curvature of the antrum as seen in our patient is the commonest location [1]. Most patients are either asymptomatic or report nonspecific upper gastrointestinal symptoms as our patient, although bleeding, mechanical obstruction, acute pancreatitis and malignant transformation have all been described as complications [[9], [10], [11]]. Distinction from other submucosal tumours can be challenging. A retrospective radiological study highlighted the following characteristics favouring ectopic pancreas over GIST and leiomyoma: prepyloric or duodenal location, endoluminal growth pattern, ill-defined borders, prominent enhancement of overlying mucosa and a long diameter to short diameter ratio greater than 1.4 [12]. In our case, the location and long-to-short diameter ratio of 1.7 favoured ectopic pancreas, while other features supported the diagnosis of GIST/leiomyoma. An earlier endoscopic study identified the tumour location and size smaller than 3 cm as factors supporting the diagnosis of ectopic pancreas. Histologically, the diagnosis of ectopic pancreas is straightforward when all constituents of a normal pancreas are present. After negative surface biopsies in our case endoscopic ultrasonography with fine-needle aspiration biopsy (EUS-FNA) was considered. Based on discussion at a multidisciplinary meeting at our institution, we opted to confirm the diagnosis surgically. This decision was based on the mediocre diagnostic yield of EUS-FNA for tumours smaller than 2 cm [3]. GISTs are the commonest mesenchymal tumours of the gastrointestinal tract, occurring anywhere along the gastrointestinal tract, with almost two thirds in the stomach and 30% in the small bowel [7].

Large GISTs have irregular margins, areas of necrosis and are ulcerated. About 50% of patients with GISTs have metastases in the liver or peritoneum [13]. GISTs, smaller than 3 cm, are particularly difficult to distinguish from other submucosal tumours as they are well-defined and homogenous. Histologically, it is a spindle-celled tumour and up to 90% are c-kit (CD117) positive on immunohistochemistry [7].

The standard treatment for localized GISTs is complete R0 surgical excision, avoiding tumour rupture, and without dissection of clinically-negative lymph nodes [14]. Although the feasibility and safety of the laparoscopic approach for GIST resection has been demonstrated in many retrospective studies [15], laparoscopic surgery is suggested only for GISTs favourably located in the greater curvature or anterior wall of the stomach [16]. If unfavourably located, there is a risk of stenosis of the lumen postoperatively, and guaranteed R0 resection is still difficult with laparoscopic procedures. The feasibility, safety and oncological outcome of this technique for unfavourably located GISTs remain unclear [17]. Endoscopic resection is an emerging treatment modality for GIST, using endoscopic band ligation (EBL), endoscopic submucosal dissection (ESD) [18], endoscopic submucosal excavation (ESE), endoscopic full-thickness resection (EFTR) and submucosal tunneling endoscopic resection (STER) [19]. The wide adoption of these techniques is currently hampered by the relatively high perforation risk which could lead to peritoneal seeding and uncertainty regarding the rate of R0 resection [20].

## 4 Conclusion

It is often difficult to determine the aetiology of submucosal gastric lesions based on clinical, endoscopic and imaging findings. Despite using multiple diagnostic modalities, the diagnosis is often ultimately proven at surgery [21,22].

## Conflict of interest

Neither ZŠ nor JH have any conflict of interest, relevant to the submitted contribution.

## Funding source

The research, presented in the submitted contribution, was not funded.

## Ethical approval

Case reports are exempt from ethical approval in our institution. The patient has provided written informed consent for the publication of this anonymised case report.

## Consent

Written informed consent was obtained from the patient for publication of this case report and accompanying images. A copy of the written consent is available for review by the Editor-in-Chief of this journal on request.

## Author contribution

ZŠ conceived the study and collected the data.

JH wrote the paper.

Both authors critically reviewed the manuscript and approved the final submitted version.

## Registration of research studies

The submitted contribution is a case report that was not registered.

## Guarantor

Zdravko Štor.

## Provenance and peer review

Not commissioned, externally peer reviewed.
